# Base Flipping in Open Complex Formation at Bacterial Promoters

**DOI:** 10.3390/biom5020668

**Published:** 2015-04-28

**Authors:** Mary E. Karpen, Pieter L. deHaseth

**Affiliations:** 1 Department of Chemistry, Grand Valley State University, 1 Campus Drive, 312 Padnos Hall, Allendale, MI 49401, USA; E-Mail: karpenm@gvsu.edu; 2 Center for RNA Molecular Biology, Case Western Reserve University, 2109 Adelbert Road, Cleveland, OH 44106, USA; 3 Department of Biochemistry, Case Western Reserve University, 2109 Adelbert Road, Cleveland, OH 44106, USA

**Keywords:** RNA polymerase, base flipping, promoter strand separation, open complex

## Abstract

In the process of transcription initiation, the bacterial RNA polymerase binds double-stranded (ds) promoter DNA and subsequently effects strand separation of 12 to 14 base pairs (bp), including the start site of transcription, to form the so-called “open complex” (also referred to as RP_o_). This complex is competent to initiate RNA synthesis. Here we will review the role of σ^70^ and its homologs in the strand separation process, and evidence that strand separation is initiated at the −11A (the A of the non-template strand that is 11 bp upstream from the transcription start site) of the promoter. By using the fluorescent adenine analog, 2-aminopurine, it was demonstrated that the −11A on the non-template strand flips out of the DNA helix and into a hydrophobic pocket where it stacks with tyrosine 430 of σ^70^. Open complexes are remarkably stable, even though* in vivo*, and under most experimental conditions* in vitro*, dsDNA is much more stable than its strand-separated form. Subsequent structural studies of other researchers have confirmed that in the open complex the −11A has flipped into a hydrophobic pocket of σ^70^. It was also revealed that RP_o_ was stabilized by three additional bases of the non-template strand being flipped out of the helix and into hydrophobic pockets, further preventing re-annealing of the two complementary DNA strands.

## 1. Introduction

**Figure 1:**
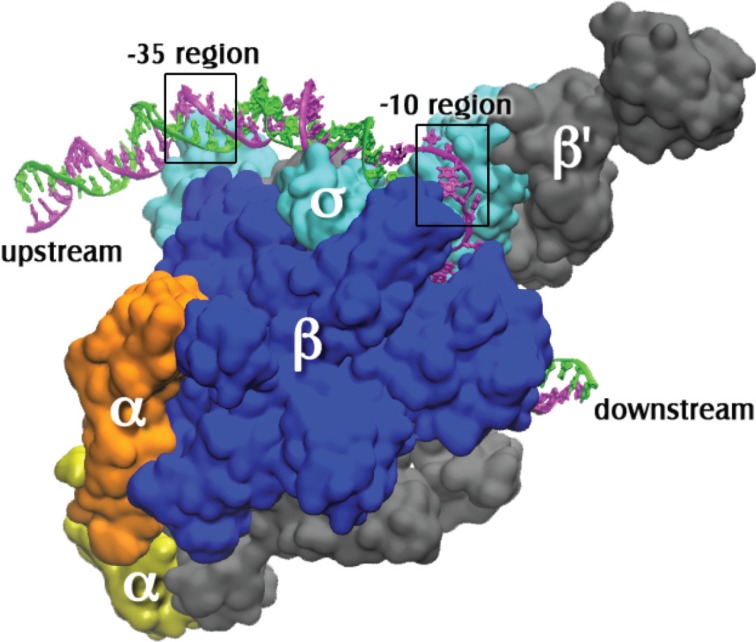
The open complex of RNA polymerase holoenzyme with promoter DNA. The coordinates for the protein and the DNA bases from −12 to +12 of the nontemplate strand (magenta) and −4 to +12 of the template strand (green) are from PDB X‑ray crystal coordinates 4G7O from *Thermus thermophilus* open complex as reported by Zhang* et al.* [8]. The sigma subunit is σ^A^. Additional upstream and downstream DNA coordinates were modeled using the electron microscopy coordinates from PDB entry 3IYD [9]. The holoenzyme subunits are labeled and the precise locations of the −10 (on the nontemplate strand) and the −35 (on ds promoter DNA) regions are indicated by boxes.

The bacterial transcription apparatus is simple in comparison to that of eukaryotes or archaea. There is one type of RNA polymerase (RNAP), which is referred to as the “core” RNAP. It typically has five subunits: α2, β, β', and ω [1]. The core RNAP has striking sequence and structural resemblances to the eukaryotic and archaeal RNA polymerases [2,3,4]. In order for RNAP bind to a promoter and initiate transcription, a “sigma” transcription initiation factor is needed (see [5] for a recent review). The sigma factor first binds to the RNAP; the complex of RNAP and sigma factor is referred to as the “holo” RNAP, or also (as in this review) just RNAP (Figure 1). Interestingly, there are also pronounced similarities between the structures of the bacterial holo RNAP and the complex of the TFIIB transcription factor and Pol II RNA polymerase of eukaryotes [6]. Many bacteria have an arsenal of various sigma factors, e.g., *E. coli* has 7, *B. subtilis* has 18 and *S. coelicolor* has 63 [7]. Each sigma factor guides RNAP to a specific set of promoters and thus drives expression of particular genes. In this manner, different sigma factors could, for example, aid the bacterial cell in dealing with different types of stress [5].

The double stranded DNA site that specifically binds RNAP is called a promoter. A typical bacterial promoter has several sequence-specific regions that are contacted by RNAP. These regions include the −35 and −10 hexamers (consensus sequences TTGACA and TATAAT, respectively), which are respectively 35 base pairs (bp) and 10 bp upstream from the transcription start site, designated +1 (Figure 1). Another important region of contact is a stretch of Gs immediately downstream of the −10 hexamer. All these features are not found in all promoters, but in general it is true that the more of them a promoter possesses, the faster it is in RNAP binding. This does not imply that initiation of RNA synthesis is then faster as well; consensus promoters may actually be slower due to poor promoter clearance (e.g., see [10]).

**Figure 2:**
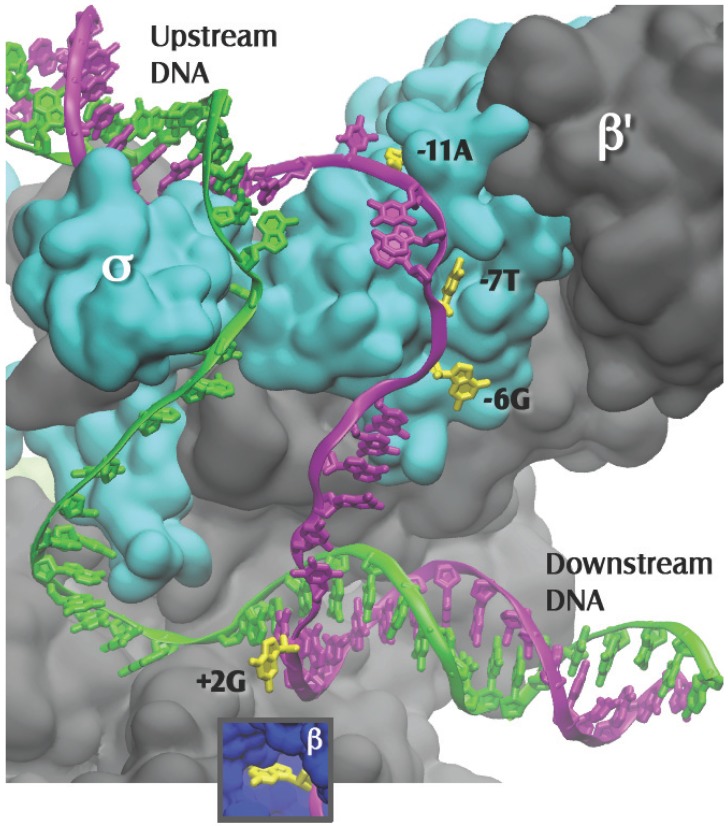
Close up of Figure 1, with the β subunit removed to reveal the transcription bubble and the flipped bases in their pockets. Template DNA is in green and nontemplate DNA is in magenta, with the flipped-out bases in yellow. Bases −11A and −7T interact solely with the σ subunit. Base −6G is at the σ-β subunit interface. Base +2G interacts solely with the β subunit (insert). The −12T nontemplate base is shown in the figure as unpaired, as it is in the 4G7O coordinate set; it is likely base paired in the native promoter.

The sigma factor of RNAP is involved both in promoter recognition and in promoter strand separation. When RNAP first binds to promoter DNA, a “closed complex” is formed in which the promoter remains double stranded. Several additional intermediate complexes then form, with conformational changes in both the RNAP and the promoter, resulting in the “open complex” (RP_o_). In this complex 12–14 base pairs have been disrupted [11,12,13], enabling the template strand to reach the active site of the RNAP [8,14] (Figure 2). Here the template DNA pairs with the incoming substrate nucleotide triphosphates [15], thus programming the sequence of nucleotides in the newly synthesized RNA. This review focuses on the mechanism of formation of the open complex, as well as its structure.

## 2. History

To the best of our knowledge, the terms “closed complex” and “open complex” were first used in 1974 by Chamberlin in a review article [16]. Evidence for strand separation at that time included the temperature-dependence of the initiation of RNA synthesis, interpreted as a “localized denaturation event”. Strong additional evidence was provided by Saucier and Wang [17], who used a sensitive method for detecting DNA strand separation, based on the effect it has on supercoiling of plasmid DNA. The advent of chemical probes such as dimethyl sulphate and KMnO_4_ for monitoring DNA strand separation [12,13,18] made it possible to determine the region of strand separation with single bp resolution. For most bacterial promoters, DNA strand separation was found to occur from the −11A in the −10 region through the +2 base just downstream of the RNA synthesis start site (e.g., [19]).

In 1988, when it was not yet clear which subunits of the bacterial RNAP were involved in the strand separation process, Helmann and Chamberlin postulated in a review article that the sigma factor was the responsible subunit. The basis for this hypothesis was the presence of a stretch of highly conserved aromatic amino acids in region 2.3 of *E. coli* σ^70^ [20,21] and *B. subtilis* σ^A^ [22], which was thought to play a role similar to the aromatic amino acids in single stranded (ss) RNA or DNA binding proteins. This was a plausible suggestion as an adjacent stretch of σ^70^, region 2.4, had previously been shown to be involved in recognition of the −12 T-A bp of the −10 hexamer. As first experimentally shown by Helmann and co-workers for *B. subtilis *σ^A^ [22], and later demonstrated in detail for σ^70^ of *E. coli* [23,24,25], the region 2.3 aromatic amino acids were indeed found to play an important role in RP_o_ formation. Especially Y425, Y430, W433 and W434 (*E. coli* σ^70^ numbering; Y is tyrosine and W, tryptophan), highly conserved in bacterial strains, were found to be important for the strand-separation process [1]. The high extent of sequence conservation makes it possible to directly compare structure and function results obtained across bacterial species.

## 3. The Closed Complex and Other Intermediates

This topic has recently been reviewed in detail by Saecker* et al.* 2011 [1]. Studies of the lambda P_R_ promoter indicate that there are minimally three intermediates on the way to the open complex: the closed complex, RP_c_ and two other intermediates, which have been named I_1_ and I_2_. A scheme for open complex formation would be as follows (R is RNAP and P, promoter DNA):

(1) $$\text{R}+P⇌{\text{RP}}_{c}⇌I_{1}⇌I_{2}⇌{\text{RP}}_{O}$$

The closed complex lacks DNA strand separation (no reaction with MnO_4_^−^, which targets thymines (T) in strand-separated regions of DNA), is unstable (*i.e.*, it readily dissociates to the free RNAP and promoter DNA) and features protection of promoter DNA from DNase I cutting from −38 through +1. The intermediate, I_1_, also lacks strand separation, but it has an extended downstream region of protection from DNase I, reaching approximately to +20. The rate-limiting step in RP_o_ formation is the conversion of I_1_ to I_2_, while in the reverse direction the same step (now I_2_ to I_1_) is limiting as well. The I_1_ to I_2_ conversion is highly temperature dependent, as expected for strand opening [1]. Finally, the I_2_ to RP_o_ step is thought to involve major conformational re-arrangements in the RNAP. The Saecker* et al.* review [1] also considers an additional intermediate, I_3_, which would occur after the rate-limiting step.

## 4. The Importance of −11A for Promoter DNA Melting

The −11A and −7T bases of the promoter −10 region (consensus sequence in the non-template strand −12TATAAT-7) are the most highly conserved [1]. The −11 A-T bp is also the most upstream strand-separated bp for most promoters. For this reason it had been thought that promoter strand separation might commence there [26,27,28,29,30]. The model was that the −11A would flip out of the DNA helix and into a hydrophobic pocket of the RNA polymerase, where the specific interactions with RP_o_ would be established. It was found that the N1 [31] and an unsubstitued C2 hydrogen [32] of the −11A purine ring were important for strand separation, likely because they support establishment of such interactions. Fenton and Gralla [23] in 2000 speculated that both Y430 and W433 would stack with −11A in sandwich fashion, being correct with respect to the Y430.

Experimental evidence for an important role of the −11A in RP_o_ formation was obtained by its substitution with the A analog 2-aminopurine (2-AP) [29]. Importantly, the promoter used in this experiment had two non-consensus bases in the −10 region, as well as other features that made it a “weak” promoter. When adenine was in the −11 position, this promoter showed strand separation upon RNAP binding. With 2-AP in the −11 position however, in the presence of RNAP, promoter strand separation was not detected, either at −11 or at any downstream bases. For this reason the −11A was dubbed the “master” base in controlling strand separation [29]. Another experiment took advantage of the fluorescence properties of 2-AP. Using a consensus model DNA with a truncated −10 hexamer consisting of just the −12 T-A base pair and an overhanging −11 2-AP, it was found that upon addition of RNAP, the mobility of the −11 2-AP was greatly decreased, while the spectral characteristics indicated that it was now in a more hydrophobic environment [30]. This is the expected result for a −11 2-AP that had been flipped out of ds DNA and into a hydrophobic pocket of σ^70^.

Strong additional support for −11A flipping was again obtained by substituting the −11 position with a 2-AP, now in a promoter designed to have an optimal sequence, so that the presence of a −11 2-AP did not inhibit strand separation. The expectation was that RNAP, in orchestrating promoter DNA melting, would unstack the 2-AP from its two neighboring bases, preventing quenching of the −11 2-AP fluorescence and thus eliciting a greatly enhanced fluorescence signal. Such enhancement of fluorescence had been seen before with *E. coli* promoters containing 2-AP at various other positions in the region of RNAP-dependent strand separation [33,34]; see also [35]. In contrast, in this experiment, the enhancement of the −11 2-AP fluorescence was barely detectable. A large enhancement of the fluorescence signal was observed, however, when mutant RNAP with a Y430A substitution in σ^70^ was used [36]. This result was interpreted to indicate that wild type RNAP would flip the −11 2-AP out of the helix and into a pocket where it now could stack with Y430, which again quenched its fluorescence. This approach was analogous to those demonstrating base flipping by methyltransferase [37] or restriction endonucleases [38].

## 5. Flipping of Other Bases of the Non-Template Strand

Prior to the availability of high resolution structures, the inference was made that in the steps leading to RP_o_ formation, the −7T was flipped out of the ds DNA as well. Based on knowledge of how RNAP interacted with the −35 region [21], Shultzaberger* et al.* deduced that σ^70^ would face the −11A base in the major groove, and the −7T in the minor groove [39]. They reasoned that interactions in the minor groove alone could not have achieved the very high conservation observed for the −7T. Thus, by necessity, −7T recognition must have taken place subsequent to its removal out of the ds DNA, and upon its insertion into a pocket of σ^70^ [39].

The first high resolution structure that shed light on the recognition of the bases in the nontemplate (NT) strand of the −10 region was obtained by Feklistov and Darst in 2011. They crystallized a complex composed of a fragment of *T. aquaticus* σ^A^ [40], including regions 1.2 through 2.4, and ss NT DNA. The NT DNA strand was from −14 through −4, but for technical reasons, contacts to −6G, −5G and −4G were unable to be discerned. This structure confirmed the prior experimental and theoretical evidence shown above for, respectively, the −11A [36] and −7T [39] flipping during open complex formation (Figure 2). Interestingly, the −11A was found to fit very tightly in its pocket, while the pocket for the −7T was more spacious, but not enough so to accommodate a purine [40].

In the later Zhang* et al.* structure [8], the *T. thermophilus* holoenzyme was complexed to an elaborate model promoter with upstream ss NT and template DNA, and downstream ds DNA. In addition to the −11A and −7T, now two other flipped-out bases in their hydrophobic pockets, −6G and +2G, were revealed [8] (Figure 2). Flipped bases have many available groups that allow them to readily form multiple contacts in their pockets, establishing very stable interactions with the RNAP. Thus, at a gene’s promoter, the four NT flipped bases, one at either end of the strand separated region (−11A and +2G) and two near the middle (−7T and −6G), would stabilize the RP_o_ by preventing re-annealing of the strands under the cellular conditions, which favor ds DNA over the separate single strands. It was recently found that strand separation at an *E. coli* σ^E^ promoter was also initiated by base flipping; here the highly conserved -10C was moved out of the ds promoter DNA and into a pocket of the sigma factor σ^E^ [41]. Consequently it is possible that promoter strand separation occurs by similar mechanisms regardless of the sigma factor that is bound to the core RNAP.

## 6. Mechanism of Strand Opening

RNAP must specifically bind and melt the promoter’s −10 region. As observed by Roberts and Roberts almost 20 years ago [42], and further elaborated by Fenton and Gralla [43] and Feklistov and Darst [40], RNAP recognizes the −10 region bases from −11 through −7 in their ss form. This RNAP-DNA binding energy offsets the energy required for DNA melting, thus facilitating strand separation. The recognition of the −12 A-T bp was found to be stimulated by the interaction of RNAP with other NT bases of the −10 element [44]. Similarly, it was observed that recognition of the −12 A-T in its ds form by σ^70^ Q437 required prior flipping of the −11A [40].

It is likely that the σ^70^ W433 side chain functions as a wedge that promotes −11A flipping (an old hypothesis [25]), as in the open complex W433 is seen to occupy the position where the −11A was prior to its flipping [8,40]. It is tempting to interpret this as evidence for an active role of the RNAP in −11A base flipping and thus also in promoter DNA melting. However, it was pointed out that the role of the W433 may also be to prevent the −11A from returning back into the helix [40], which would be more akin to a passive role, with base flipping occurring due to thermal motions, and RNAP stabilizing the flipped state. Thus, whether RNAP-dependent DNA strand separation involves an active or a passive mechanism remains unresolved.

There is no consensus regarding the mechanism of strand opening. A plausible model, mentioned above, is that the flipping of the −11A would initiate the process, and the ds DNA would subsequently be unzipped in downstream direction. The recognition of the −7T would happen upon arrival in its pocket, but the kinetic relationship between DNA unzipping and −11A, −7T, −6G or +2G flipping has not yet been determined. In a recent paper, Heyduk and Heyduk showed that the −7T may play a greater role in the initiation of promoter DNA melting than previously thought [45]. The presumed upstream to downstream direction of strand separation is consistent with several observations. These include the results of Brownian dynamics simulations [46], a mutant RNA polymerase which was only able to melt a promoter from −11 through −7 [47], the finding that upstream DNA is melted prior to downstream DNA [36,48,49,50], and the effect of substitution of the “master base” mentioned above [29] (see also [27]). However, progressive upstream to downstream melting has not been observed directly in kinetic experiments. It may be that propagation of melting is too fast to be observed by currently available methods or that strand separation indeed does not take place in an upstream to downstream order [33], for example because the whole region might strand separate at once [51].

## 7. Structural and Functional Properties of Open Complexes

The NT strand follows the contour of the RNAP surface, resulting in a sharp bend between the flipped −11A and −7T, with σ^70^ T429 [52] acting as a fulcrum [40]. The path of the template strand had previously been modeled to lead to the active site [14]. The more recent structure shows that the template strand follows a gradual curvature around σ^70^ region 3.2, (the sigma “finger” [8]) (Figure 2). In the final stages of open complex formation, subsequent to conformational changes in the RNAP holoenzyme, interactions of the β and β’ RNAP core subunits with ss and ds DNA are established [1,8,53]. These interactions include those between the β subunit and the ss DNA core recognition element (the downstream ss NT DNA from −4 to +2) [8], as well as further downstream interactions largely between the β' and ds DNA [1,8,53].

The size of the transcription bubble has been found to be dynamic; the region that is single stranded has been found to fluctuate in the millisecond timescale [54]. This may affect the choice of the template strand nucleotide at which transcription initiates. In addition, the open complex resembles a transcription elongation complex in terms of the position of the RNAP clamp, which is closed for both [8]. Interestingly there is an additional similarity in the structures of the template strand. In the open complex the template strand is already organized to look like it would in the ds A-form helix that is established for the hybrid of the template strand DNA with the newly synthesized RNA [8] (Figure 2). Thus the RNAP-promoter open complex is well-prepared to initiate RNA synthesis, as pointed out by Zhang* et al.* [8].

## 8. Conclusions

The first step in open complex formation is the interaction of the promoter, in its double stranded form, with the sigma factor of RNAP. The −35 region remains double stranded, and the nontemplate −11 base flips away from its base pair and into a pocket on the surface of the sigma factor. This base flipping event initiates the melting process required to form the transcription bubble. The bubble grows in an upstream to downstream direction and is stabilized by additional flipped bases of the non-template strand. Interactions of these flipped bases with RNAP’s sigma subunit and/or beta subunit are established; these promoter-RNAP interactions with the non-template strand stabilize the transcription bubble, allowing the single stranded template DNA to program RNA synthesis at the RNAP active site.
